# Formation of a Western Society of Dental Surgeons Contemplated

**Published:** 1842-12

**Authors:** 


*Formation of a Western Society of Dental Surgeons contemplated.*—We are informed by a professional friend, of Cincinnati, that the formation of a Society of Dental Surgeons west of the Alleghany mountains, is contemplated. We have long expected that a move of this sort would be made, and we wish those who may be engaged in the praiseworthy undertaking, every success. If it be rightly organized and properly conducted, the interest of the profession of the west, as well as the good of community generally, will be greatly advanced by it. Among the dentists of the west, we know that there are men of talent and science, and we should be gratified to see them exerting themselves to elevate the character of the profession.

